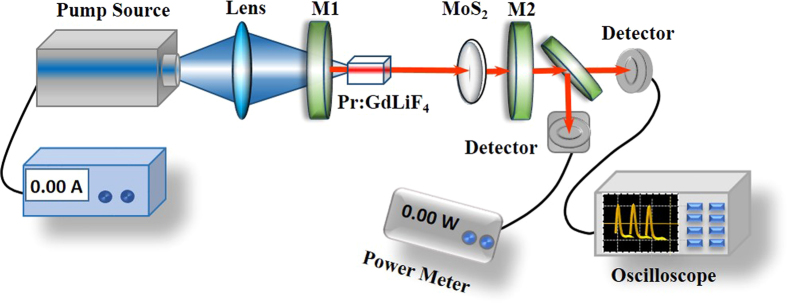# Atomic-layer molybdenum sulfide optical modulator for visible coherent light

**DOI:** 10.1038/srep11342

**Published:** 2015-06-12

**Authors:** Yuxia Zhang, Shuxian Wang, Haohai Yu, Huaijin Zhang, Yanxue Chen, Liangmo Mei, Alberto Di Lieto, Mauro Tonelli, Jiyang Wang

**Affiliations:** 1 State Key Laboratory of Crystal Materials and Institute of Crystal Materials, Shandong University, Jinan 250100, China; 2 School of Physics, Shandong University, Jinan 250100, China; 3 NEST Istituto Nanoscienze-CNR and Dipartimento di Fisica dell’Università di Pisa, Largo B. Pontecorvo 3, 56127 Pisa, Italy

## Abstract

Coherent light sources in the visible range are playing important roles in our daily life and modern technology, since about 50% of the capability of the our human brains is devoted to processing visual information. Visible lasers can be achieved by nonlinear optical process of infrared lasers and direct lasing of gain materials, and the latter has advantages in the aspects of compactness, efficiency, simplicity, etc. However, due to lack of visible optical modulators, the directly generated visible lasers with only a gain material are constrained in continuous-wave operation. Here, we demonstrated the fabrication of a visible optical modulator and pulsed visible lasers based on atomic-layer molybdenum sulfide (MoS_2_), a ultrathin two-dimensional material with about 9–10 layers. By employing the nonlinear absorption of the modulator, the pulsed orange, red and deep red lasers were directly generated. Besides, the present atomic-layer MoS_2_ optical modulator has broadband modulating properties and advantages in the simple preparation process. The present results experimentally verify the theoretical prediction for the low-dimensional optoelectronic modulating devices in the visible wavelength region and may open an attractive avenue for removing a stumbling block for the further development of pulsed visible lasers.

Visual information from our eyes goes to our brains and then is processed^1^,^2^,^3^. It has been well-understood that nearly 50% of the capability of our brains is devoted to processing visual information^2^,^3^. Therefore, besides the numerous applications in our daily life, including entertainment, education and medical treatment, visible lasers also have important requirements in modern science and technologies^1^,^7^. Nonlinear optics and direct lasing of gain materials^4^,^5^,^6^ are the techniques for getting the visible lasers, beside the visible laser diodes which have relatively poor coherence^8^,^9^. In contrast to lasers achieved by nonlinear frequency-shifting of infrared lasers^10^, direct lasing has the advantages in the aspects of compactness, efficiency, simplicity, etc. In the laser regime, pulsed lasers with fast- or ultrafast pulses and high- or ultra-high peak power provide a platform for the studying ultrafast kinetics process^11^,^12^, realizing strong field physics^13^,^14^, etc., and is an vital scientific topic in lasers. A optical modulator for tuning the gain or loss in the oscillator is a crucial device for the generation of pulses. However, constrained by optical modulators, especially passive optical modulators, the directly generated visible pulses are rare up to now^15^,^16^. In theory, the optical modulators in visible lasers can be realized by semiconductor saturable absorber mirrors^17^, but they require complexly and elaborately designed quantum wells, besides their narrow response wavelength band.

Recent years, atomic-layered transition-metal dichalcogenides have emerged as promising next-generation optoelectronic materials, since their exceptional and interesting electronic and photonic properties^18^,^19^,^20^,^21^,^22^,^23^. Their strong nonlinear optical response, including the second and third nonlinearities are also remarkable^21^,^24^. Based on the nonlinear absorption and band-gap engineering, the transition-metal dichalcogenides saturable absorbers were developed as broadband optical modulators for pulsed laser operation in the wavelength range from 1 μm to 2 μm^21^,^25^. However, maybe associated to the large photon energy carried by the visible laser and the band-gap of present low-dimensional optoelectronic materials, the applications of low-dimensional optoelectronic materials, including, carbon nanotubes, graphene, topological insulators and transition metal dichalcogenides, in visible lasers as a modulating device mainly exist in theoretical prediction^21^,^26^,^27^,^28^,^29^. Considering the nonlinear optical properties of the low-dimensional optoelectronic materials, few-layered MoS_2_ has large nonlinearity and saturable intensity, 5 orders of magnitude larger than graphene in the visible wavelength range^21^,^25^,^27^, which theoretically indicates that few-layered MoS_2_ should be a promising optical modulator for the generation of pulsed visible laser with large pulse energy. Here, we report optical modulation of visible coherent light by less than 10 layers MoS_2_ in the wavelength band from orange to deep red. Based on the visible MoS_2_ optical modulator, the directly generated pulsed visible lasers were demonstrated.

## Results

### Raman, atomic force microscopy and spectral analysis of MoS_2_ film

**Figure 1:**
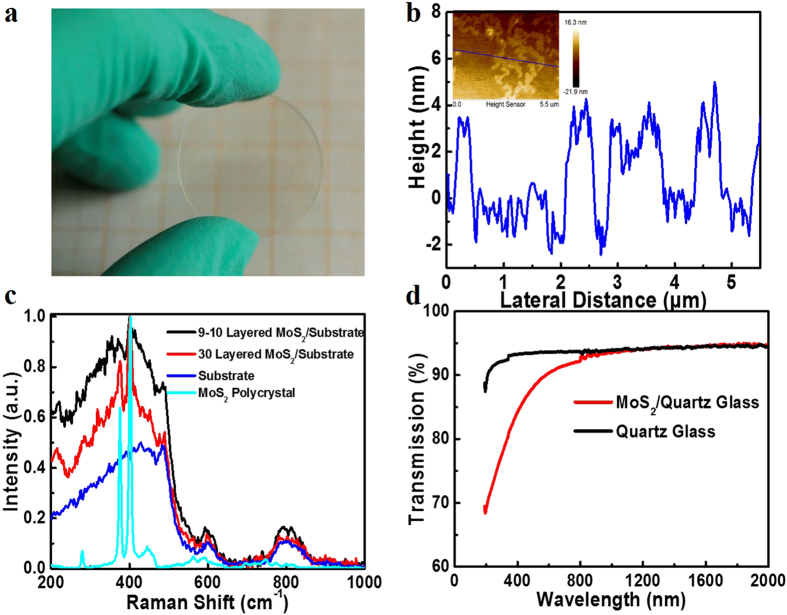
Characterization of prepared MoS2 thin film. (**a**) Prepared 9–10 layered MoS_2_ sample on a quartz glass substrate with the dimensions of *Φ* 25 mm × 1 mm (*Φ* is the wafer diameter). (**b**) Typical variation of MoS_2_ thin film height. Inset: Morphology of sand-abraded MoS_2_ thin film measured with atomic force microcopy. (**c**) Raman spectra of prepared 9–10 layered MoS_2_/quartz glass sample, 30 layered MoS_2_/quartz glass sample, quartz substrate, and polycrystalline MoS_2_. (**d**) Absorption spectra of prepared MoS_2_/quartz glass sample and quartz glass substrate.

The MoS_2_ film was prepared with pulsed laser deposition (PLD) with a polished optical-grade far-ultraviolet quartz glass wafer (*Φ* 25 mm × 1 mm, *Φ* is the wafer diameter) which can provide high resolution in the visible range^21^. The prepared sample is shown in Fig. 1a. In order to accurately measure the number of layers, the prepared MoS_2_ samples were investigated with atomic force microscopy (AFM) by abrading with sand. The change of the MoS_2_ height is provided in Fig. 1b which shows that the height change is about 5.8 nm and the MoS_2_ film is about 9–10 layers on the substrate since the MoS_2_ layers were assumed to bond together via the Van der Waals’ interaction^30^. The 9–10 layered MoS_2_ sample was characterized with a Raman spectrometer and the Raman shifting is displayed in Fig. 1c. For comparison, the 30 layered MoS_2_ thin film and polycrystalline MoS_2_ are also presented in this figure. From this figure, it can found that for the 9–10 layered MoS_2_ thin film, an in-plane E_2g_^1^ vibrational mode and an out-of-plane A_1g_ vibrational mode appear at 378 cm^−1^ and 400 cm^−1^, respectively. The A_1g_ mode also shows a shift to larger wavenumbers with the increase of layers, which is in agreement with the previous reported anomalous lattice vibrations of layered MoS_2_^31^.

For determining the responding wavelength range of the prepared MoS_2_ sample, the transmission spectrum was measured with a V-570 JASCO UV/VIS/NIR spectrophotometer from the wavelength of 190 nm and is shown in Fig. 1d. For comparison, the spectrum of a quartz glass substrate is also given. From this figure, it can be observed that the transmission increases with the increase of the wavelength, changes smooth when the wavelength is larger than 1000 nm and displays the prepared sample becoming indistinguishable with the substrate when the wavelength is larger than 1250 nm. The results indicate that the band gap of the prepared sample is about 1 eV in the range of 0.86–1.29 eV of multilayered MoS_2_^32^,^33^ and also in agreement with the theoretical calculation of about 9–10 layered MoS_2_^34^. It should be noted that in the visible range from 380 nm to 780 nm, the transmission of the prepared MoS_2_/quartz glass sample increases from 83.2% to 92.1% with augmenting the wavelength. Therefore, we can get the conclusion that all the visible photons can transfer the electrons from the valence band to the conduction band of the prepared sample and the prepared MoS_2_ sample has response in all the visible range.

Based on the measurement shown in Fig. 1 and the previous reports on the saturable absorption properties of few- and multi-layered MoS_2_^21^,^25^, it can be gotten the conclusion that under strong excitation with the wavelength shorter than 1250 nm, the electronic states could be fully occupied due to the transfer of electrons from the valence band to conduction band, so that the absorption of the prepared MoS_2_ thin film would be saturated. The kinetic process of absorption can be described by the power-dependent transmission formula of two-level absorber as^35^:


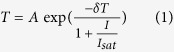


where *T* is the transmission of the MoS_2_ thin film, *A* is a normalization constant, *δT* is the absolute modulation depth of the sample, *I* is the incident intensity and *I*_*sat*_ is the saturation intensity. Considering the previous investigations on few-layered MoS_2_ thin film^21^,^25^, we can get the results that the saturation intensity *I*_*sat*_ should be in the range from 136 GW/cm^2^ to 280 GW/cm^2^ in the visible wavelength from 400 nm to 800 nm.

### Optical modulating properties of MoS_2_ film in the visible range and pulsed visible lasers

Praseodymium (Pr^3^) ions in a suitable crystalline field can emit the visible light from green to deep red wavelengths and Pr^3+^ lasers are the representative in the visible laser regime^6^. With a 1.01 at.% praseodymium doped lithium fluoride gadolinium crystal (Pr: GdLiF_4_) as the gain materials and a laser diode with a emission wavelength centered at 445 nm as the pump source, the optical modulating properties and application of the MoS_2_ sample in the laser at orange (605 nm), red (639 nm) and deep red (721 nm) wavelengths were investigated and the pulsed visible lasers were realized. In the laser experiments, different cavities were built for realizing different wavelength lasers as described in***Methods*** section and the prepared MoS_2_ thin film was used as the universal pulse modulator for the generation of all the visible laser pulses. The laser operation was controlled by inserting or removing the MoS_2_ thin film in the cavity. The continuous and Q-switched laser output power were measured by a power meter. The pulsed output performance including the repetition rate and pulse width was monitored by a digital oscilloscope and a silicon detector.

**Figure 2:**
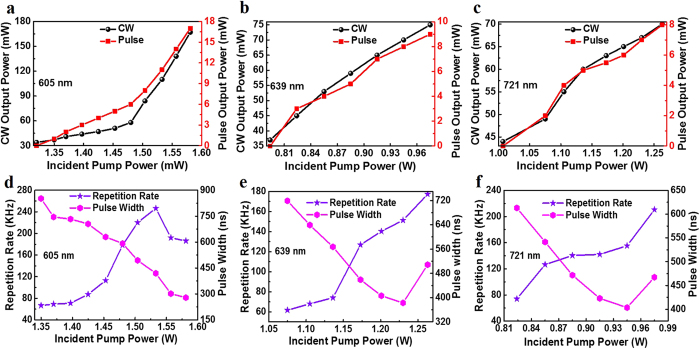
Pulsed visible laser performance with the prepared MoS2 as a modulator. (**a**)–(**c**) Continuous-wave and pulsed laser output power at the wavelength of 605 nm (orange), 639 nm (red) and 721 nm (deep red), respectively. (**d**)–(**f**) Repetition rate and pulse width of the pulsed laser at the wavelength of 605 nm (orange), 639 nm (red) and 721 nm (deep red), respectively.

By removing MoS_2_ from the cavity, the continuous-wave (CW) visible lasers were realized and the results were shown in Fig. 2a-c at the wavelengths of 605 nm, 639 nm and 721 nm, respectively. By inserting the MoS_2_ modulator, the corresponding pulsed lasers were achieved and were also presented in Fig. 2a-c for comparison with the CW lasers. From the figures shown above, we found that all the output power, including CW and pulsed lasers, increases with the increase of pump power. However, with the increase of pump power over than a threshold, the pulse performance became unstable due to the generation of CW components in the pulsed lasers which means that under this threshold, the loss modulation of the MoS_2_ thin film can switch the lasers completely based on the analysis on the passive modulating^35^. This threshold is related to the gain of the oscillating intensity in the cavity and the absorption of the modulator. Since the gain of the laser crystal and the absorption of the MoS_2_ thin film at presented wavelengths are different and the transmission of output mirrors is low (4% at 605 nm, 1.8% at 639 nm and 1.4% at 721 nm), the thresholds are changeable with the value of 1.58 W, 0.98 W and 1.26 W at the 605 nm, 639 nm and 721 nm lasers, respectively. The maximum CW output power is 248 mW, 430 mW and 232 mW, and the maximum pulsed average output power is 17 mW, 9 mW and 8 mW at the wavelength of 605 nm, 639 nm and 721 nm, respectively.

**Figure 3:**
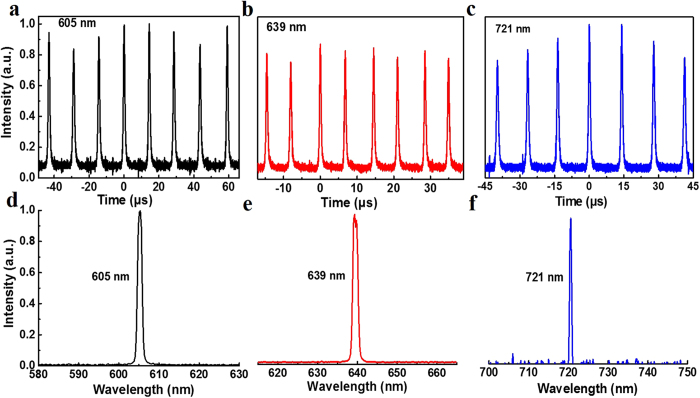
Typical pulse trains and spectra of pulsed visible lasers. (**a**)–(**c**) Pulse train with the repetition rate of 69.1 kHz at the wavelength of 605 nm, 140.3 kHz at the wavelength of 639 nm, and 74 kHz at the wavelength of 721 nm, respectively. (**d**)–(**f**) Pulsed laser spectrum with the center wavelength of 605 nm, 639 nm and 721 nm, respectively.

The repetition rate and pulse width with the increase of the pump power at different wavelength are shown in Fig. 2d–f, respectively. It should be noted that the repetition rate displays an increasing tendency with the pump power. However, the repetition rate shows a knee point under the pump power of 1.53 W at the wavelength of 605 nm, which should be related to the high-order absorption including the carrier or two-photon absorption^24^ and the high photon energy carried by this wavelength laser. The maximum repetition rate is 246 kHz, 210 kHz and 177 kHz at the wavelength of 605 nm, 639 nm and 721 nm, respectively. The pulse widths decrease with the increase of incident pump power, which is typical for the passively modulated lasers^21^,^36^. The minimum pulse width at the wavelength of 605 nm, 639 nm and 721 nm is 278 ns, 403 ns and 382 ns, respectively. With the average output power and repetition rate, the maximum pulse energy at the wavelength of 605 nm, 639 nm and 721 is 91 nJ, 52 nJ and 68 nJ, respectively. Combing the pulse energy and pulse width, the maximum peak power at the wavelength of 605 nm, 639 nm and 721 nm is 327 mW, 127 mW and 120 mW, respectively. Representative pulse trains at the wavelengths of 605 nm, 639 nm and 721 nm are shown in Fig. 3a–c, with the repetition of 69.1 kHz, 140.3 kHz, and 74 kHz, respectively. As shown in the figure, the pulses are uniformly spaced and the pulsed lasers are stable. With an optical spectrum analyzer (HR4000, Ocean Optics Inc), the pulsed laser spectra are recorded and shown in Fig. 3d–f, whose peaks are located at 605 nm, 639 nm and 721 nm, respectively.

## Discussions

From the discovery of lasers, visible lasers play important roles in many fields. However, the pulsed visible lasers are mainly achieved by the nonlinear optical process and the development of the directly generated pulsed visible lasers are constrained by rare suitable modulators. Since the first reported carbon nanotube pulsed modulator^26^, it has been theoretically predicted that low-dimension optoelectronic materials including carbon nanotube, graphene, topological insulators, and MoS_2_ could be used as pulses modulators in the visible lasers. However, up to now, no any experimental results were reported in this wavelength range. Beside strong nonlinear optical properties, MoS_2_ has a band gap in the range of 0.86 eV to 1.8 eV changeable with the layer number^34^, which means that this material should be a promising visible laser modulator. Aiming at the possible application in visible pulsed lasers, the 9–10 layered MoS_2_ thin film was prepared with the PLD method on a quartz-glass substrate. By investigating its transmission spectra and based on the previous investigation on its electronic structure and nonlinear optics^21^,^24^,^34^, we found that this prepared sample could response to the photons at the wavelength less than 1250 nm, especially in the visible range as a pulse modulator.

In order to verify the pulse modulating properties of MoS_2_ samples in the visible wavelength, the prepared sample was inserted into a simple directly generated visible laser cavity to be used as a pulse modulator. The pulsed lasers in the wavelength ranging from 605 nm to 721 nm were realized and stable pulses were achieved and analyzed, which shows that the two-dimensional MoS_2_ has excellent modulating properties in the visible range. Besides, considering the low dimension, it could be proposed that this device would also have some promising applications in the micro- and nano-optoelectronics. For future improvement of the MoS_2_ pulsed laser performance, the laser cavity and the MoS_2_ layers number should be optimized for satisfying different requirement. Furthermore, these results also experimentally verify the theoretical prediction about the applications of the two-dimensional optoelectronic materials in visible lasers as a modulator.

In conclusion, we provide an atomic-layer MoS_2_ optical modulating device for the visible lasers. The responding wavelength regime of the prepared device was investigated. Based on the device, pulsed orange, red and deep red lasers were realized which presents that the prepared device is a promising optical modulator. The results also widen the application wavelength range of two-dimensional optoelectronic materials as modulators from the well-reported infrared wavelength to the rare visible wavelength. Additionally, the present results should also be helpful for the development of lasers especially for the pulsed visible lasers which are constrained by the suitable modulator up to now. To sum up, our findings may constitute the basis for the further development of pulsed visible lasers and open a new door for the two-dimensional optoelectronic materials.

## Methods

### Preparation of atomic-layer MoS_2_ optical modulator

The atomic-layer MoS_2_ thin film was prepared by the pulsed laser deposition (PLD) technique^21^,^37^ with polycrystalline MoS_2_ powder as the raw materials. A commercial optical-grade far-ultraviolet quartz glass wafer (*Φ* 25 × 1 mm^3^) was chosen as the substrate which has high-transmission in the visible wavelength range. By cold pressing at about 70 MPa, MoS_2_ powder was pressed into a 40 mm diameter pellet. The excitation source is a Comppex Pro 201 KrF excimer laser (provided by Coherent Inc.) at a wavelength of 248 nm and a pulse width of 20 ns, which provide the radiation and ablation to the target in a spot area of about 5 mm^2^. The repetition rate of the KrF excimer laser is 5 Hz with the pulse energy of 600 mJ/pulse (corresponding to an energy density of 8.5 J/cm^2^). The base pressure of the vacuum chamber system was about 8.9 × 10^–5^ Pa, and in the deposition process, the pressure is up to about 5 × 10^–4^ Pa. Both of the MoS_2_ target and quartz-glass substrate can be rotated for ensuring the uniformity of the prepared film and the temperature of the substrate was fixed at 300 °C. The number of layers can be controlled by the deposition time.

### Laser configuration with MoS_2_ as the pulse modulator

The pulsed visible laser cavity is shown in Fig. 4. In all the visible laser experiments, a laser diode with a central wavelength of 445 nm was used as the pump source and a Pr:GdLiF_4_ crystal was employed as the gain material. The laser cavity is plane-concave formed by M1 and M2. With a f = 25 mm lens, the pump light was focused into the Pr:GdLiF_4_ crystal by passing through the input plane mirror M1, antireflection (AR) coated for the pump wavelength of 445 nm and highly reflective (HR) for the laser wavelength (550–780 nm). The gain material cut along the *a* direction has the dimensions of 2.5 mm × 2 mm × 6.9 mm (*a ×* *c* *×* *a*) and both of the end faces were polished. In order to removing the heat generated during the laser process, the gain was wrapped by an indium foil and mounted in a copper block cooled by 7 °C water. The optimized length of the cavity was optimized to be about 45 mm. By changing the output couplers M2, the orange, red and deep red lasers were obtained at the wavelengths of 605 nm, 639 nm, and 721 nm, respectively. All the output couplers M2 have the radii of curvature of 50 mm. For the orange laser, the output coupler M2 has the transmissivity of 4% at 605 nm and high transmission at 620 to 721 nm for suppressing the oscillating at red and deep red light. For the red laser, the output coupler M2 has the transmissivity of 1.8% at 639 nm and high transmission at 721 nm. For the deep red laser, the output coupler M2 has the transmissivity of 1.4% at 721 nm and high transmission at the wavelength under 700 nm. In the pulsed lasers, the MoS_2_/quartz-glass modulator was insert into the cavity as close as possible to the output coupler. The continuous and Q-switched laser output power were measured by a power meter. The pulsed output performance including the repetition rate and pulse width was monitored by a digital oscilloscope and a silicon detector.

## Additional Information

**How to cite this article**: Zhang, Y. *et al*. Atomic-layer molybdenum sulfide optical modulator for visible coherent light. *Sci. Rep*. **5**, 11342; doi: 10.1038/srep11342 (2015).

## Figures and Tables

**Figure 4:**